# Histological evaluation of symptomatic ossification of the anterior longitudinal ligament treated with etidronate disodium: a case report

**DOI:** 10.1186/s13256-016-1100-7

**Published:** 2016-11-10

**Authors:** Yusuke Sugimura, Naohisa Miyakoshi, Yuji Kasukawa, Michio Hongo, Yoichi Shimada

**Affiliations:** Department of Orthopedic Surgery, Akita University Graduate School of Medicine, 1-1-1 Hondo, Akita, 010-8543 Japan

**Keywords:** Diffuse idiopathic skeletal hyperostosis, Ossification of the anterior longitudinal ligament, Anterior cervical osteophyte, Etidronate disodium, Dysphagia

## Abstract

**Background:**

Here we report the first autopsied case involving pathological examination after two resections of symptomatic ossification of the anterior longitudinal ligament with anterior osteophytes and etidronate treatment with more than 8 years of follow-up.

**Case presentation:**

A 51-year-old Japanese man complained of severe dysphagia due to esophageal compression by ossification of his anterior longitudinal ligament with anterior cervical osteophytes. Although surgical removal of the anterior cervical osteophytes was performed following etidronate treatment (800 mg/day for 6 months), dysphagia occurred secondary to recurrent ossification of his anterior longitudinal ligament with anterior osteophytes 7 years after the initial resection. A second resection of the anterior cervical osteophytes was performed, and cyclic administration of etidronate disodium (1000 mg/day, 3-month administration and 3-month cessation) did not result in re-outgrowth of ossification of his anterior longitudinal ligament with anterior osteophytes. At 1 year and 6 months after the second surgery, he suddenly died. The pathological findings associated with the ossification of his anterior longitudinal ligament during etidronate therapy showed no recurrence of ossification of the anterior longitudinal ligament with anterior osteophytes.

**Conclusion:**

A recurrence of ossification of the anterior longitudinal ligament with anterior osteophytes formation, which caused dysphagia, was not observed with the cyclic administration of etidronate disodium at a dose of 1000 mg/day every 3 months for a period of 1 year and 5 months in the present case.

## Background

Although symptomatic ossification of the anterior longitudinal ligament (OALL) is an uncommon condition, cervical OALL in patients with diffuse idiopathic skeletal hyperostosis (DISH) has been cited as one of the most common causes of dysphagia requiring surgical management in several case reports [1]. The average 5-year postoperative course of surgical resection of ossification is generally good [2]. However, there is concern regarding recurrent cervical OALL with anterior osteophytes in patients with DISH [1]. Etidronate, a bone resorption inhibitor that is now mainly used as an antiosteoporotic medication, was historically used to prevent heterotopic ossification [3]. Its mechanism of action involves inhibition of hydroxyapatite crystal growth as shown by *in vitro* chemisorption onto the crystal surface [4]. While it is known that etidronate disodium effectively suppresses progression of ligament ossification by the absence of calcification or osteoproliferation in rats [5], no studies have reported the treatment of OALL by etidronate disodium and pathologically examined the effects of etidronate on ossification after treatment with this drug in human studies. We hypothesized that etidronate can halt the progression of anterior cervical osteophytes on OALL after surgical resection and thus avoid recurrence of dysphagia by re-outgrowth of anterior osteophytes on OALL. Here we report the first autopsied case involving pathological examination after resection of symptomatic OALL and etidronate treatment with more than 8 years of follow-up. The resection of anterior cervical osteophytes can resolve OALL-induced dysphagia or aspiration; however, whether recurrent anterior osteophytes on OALL cause symptoms over the long term remains unclear. Etidronate may be a useful medication with which to suppress enlargement of a recurrent ossification and anterior cervical osteophytes.

## Case presentation

**Fig. 1:**
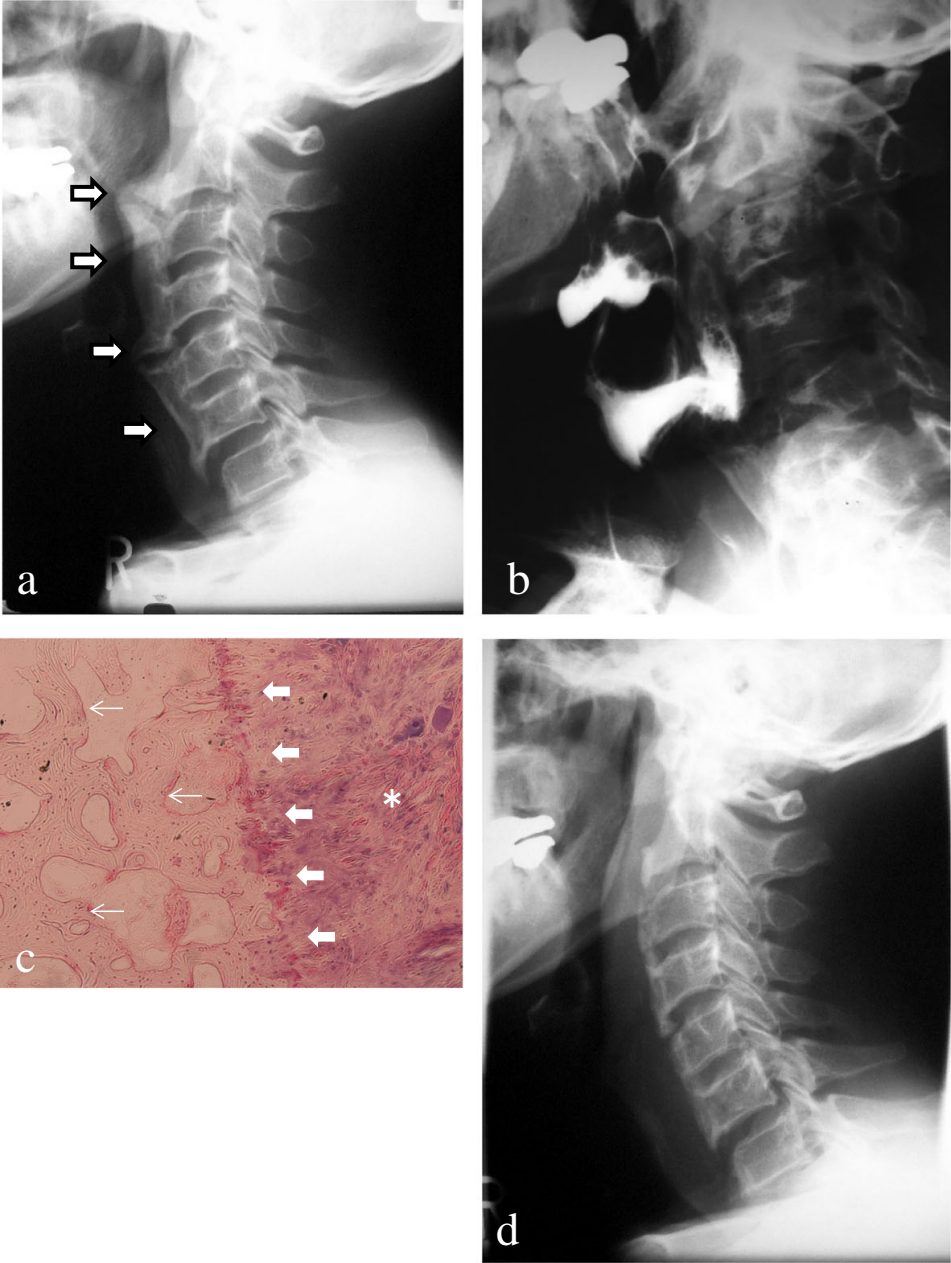
**a** Lateral radiograph at the initial presentation showed ossification of the anterior longitudinal ligament (*arrows*) from C2 to C6. **b** Lateral cervical radiograph with barium contrast revealed severe compression of the esophagus by the ossification of the anterior longitudinal ligament from C4 to C5. **c** Histopathological examination of the resected ossification revealed ossifications with a trabecular structure (*thin arrows*) from the border (*thick arrows*) of the anterior longitudinal ligament (*asterisk*). **d** Postoperative lateral radiograph 1 week after the first resection surgery of the ossification of the anterior longitudinal ligament with anterior osteophytes demonstrated decreased compression of the esophagus

A 51-year-old Japanese man presented to our hospital with a chief complaint of discomfort while swallowing. He did not have any comorbidities. An elevated mass was observed in his pharynx. The initial radiograph of his cervical spine revealed an OALL from C2 to C6 (Fig. 1a). Esophagography revealed a severe obstruction from C4 to C5 (Fig. 1b). He was diagnosed as having dysphagia due to OALL with anterior osteophytes and underwent resection of the ossification and osteophytes. Pathological examination of the resected mass showed that the ossification extended from the anterior longitudinal ligament and exhibited a trabecular structure with strut-shaped and rod-shaped components connecting each other around the bone marrow (Fig. 1c). After surgical removal of the OALL, his dysphagia resolved (Fig. 1d). He received etidronate disodium at 800 mg/day for 6 months after the bone resection to prevent postoperative recurrence of the ossification. No recurrence of ossification and osteophytes formation was found during the first postoperative year by follow-up radiographs.

**Fig. 2:**
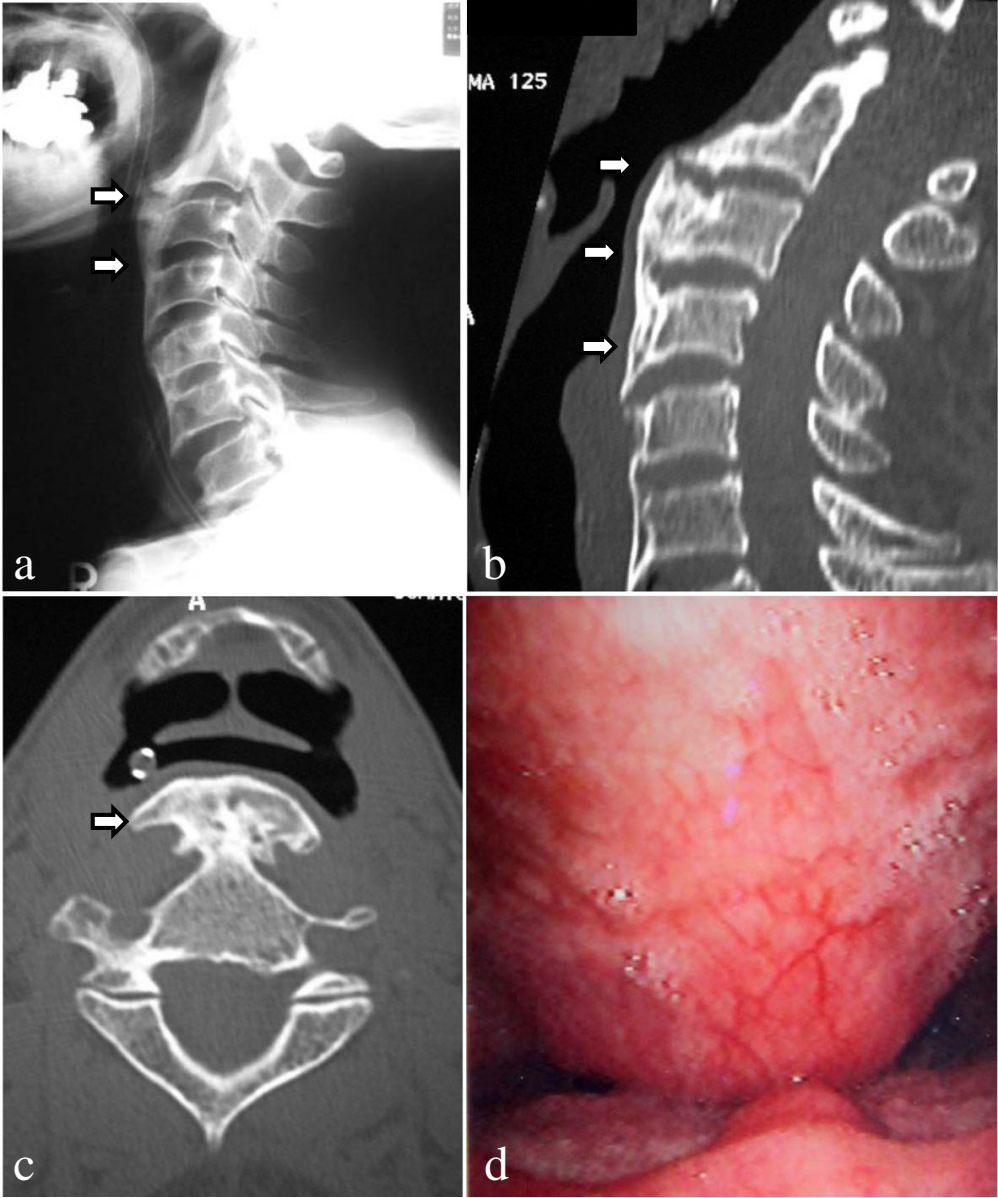
**a** A recurrent ossification of the anterior longitudinal ligament with anterior osteophytes (*arrows*) was present from C2 to C6 on a lateral radiograph taken 7 years after the first ossification of the anterior longitudinal ligament resection. **b**, **c** Sagittal and axial computed tomographic images of the cervical spine (C3, bone window) showed extensive ossification of the anterior longitudinal ligament with anterior osteophytes (*arrows*) displacing and deforming the contour of the proximal posterior pharyngeal wall. **d** Endoscopic examination revealed narrowing of the esophageal entrance by the ossification of the anterior longitudinal ligament with anterior osteophytes

**Fig. 3:**
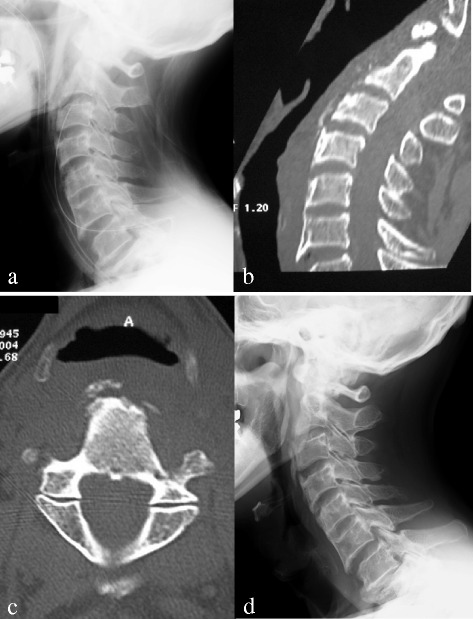
**a** Lateral radiograph after the second resection of the ossification of the anterior longitudinal ligament also demonstrated decreased compression of the esophagus. **b**, **c** Sagittal and axial computed tomographic images of the cervical spine (C3, bone window) after the second resection of the ossification of the anterior longitudinal ligament showed significantly decreased ossification and osteophytes without compression of the esophagus. **d** There was no recurrent ossification of the anterior longitudinal ligament with anterior osteophytes compressing the esophagus 1.5 years after the second resection on a lateral radiograph

However, enlargement of the ossification and osteophytes was observed 1.5 years after the surgery. His difficulty swallowing did not worsen until 5 years after resection of the ossification. He developed aspiration symptoms during the seventh postoperative year because of further enlargement of the recurrent OALL with anterior osteophytes from C2 to C6, which was revealed by a radiograph of his cervical spine (Fig. 2a). Reconstructed computed tomographic images revealed the OALL extending from C2 to C4 and compressing his hypopharynx on both sagittal (Fig. 2b) and axial (Fig. 2c) images. Narrowing of the esophageal entrance was observed on endoscopy (Fig. 2d). He was diagnosed as having dysphagia due to recurrent OALL with anterior osteophytes and underwent a second resection of the ossification and osteophytes. A postoperative radiograph showed complete resection of the OALL (Fig. 3a), and computed tomographic images also revealed removal of the OALL on both sagittal (Fig. 3b) and axial (Fig. 3c) images. His dysphagia resolved after the second surgery. He received cyclic administration of etidronate disodium at 1000 mg/day (3-month administration period followed by a 3-month unmedicated period) to prevent ossification according to a previous report [6]. After this treatment, enlargement of the ossification and osteophytes formation were not observed on radiographs (Fig. 3d).

**Fig. 4:**
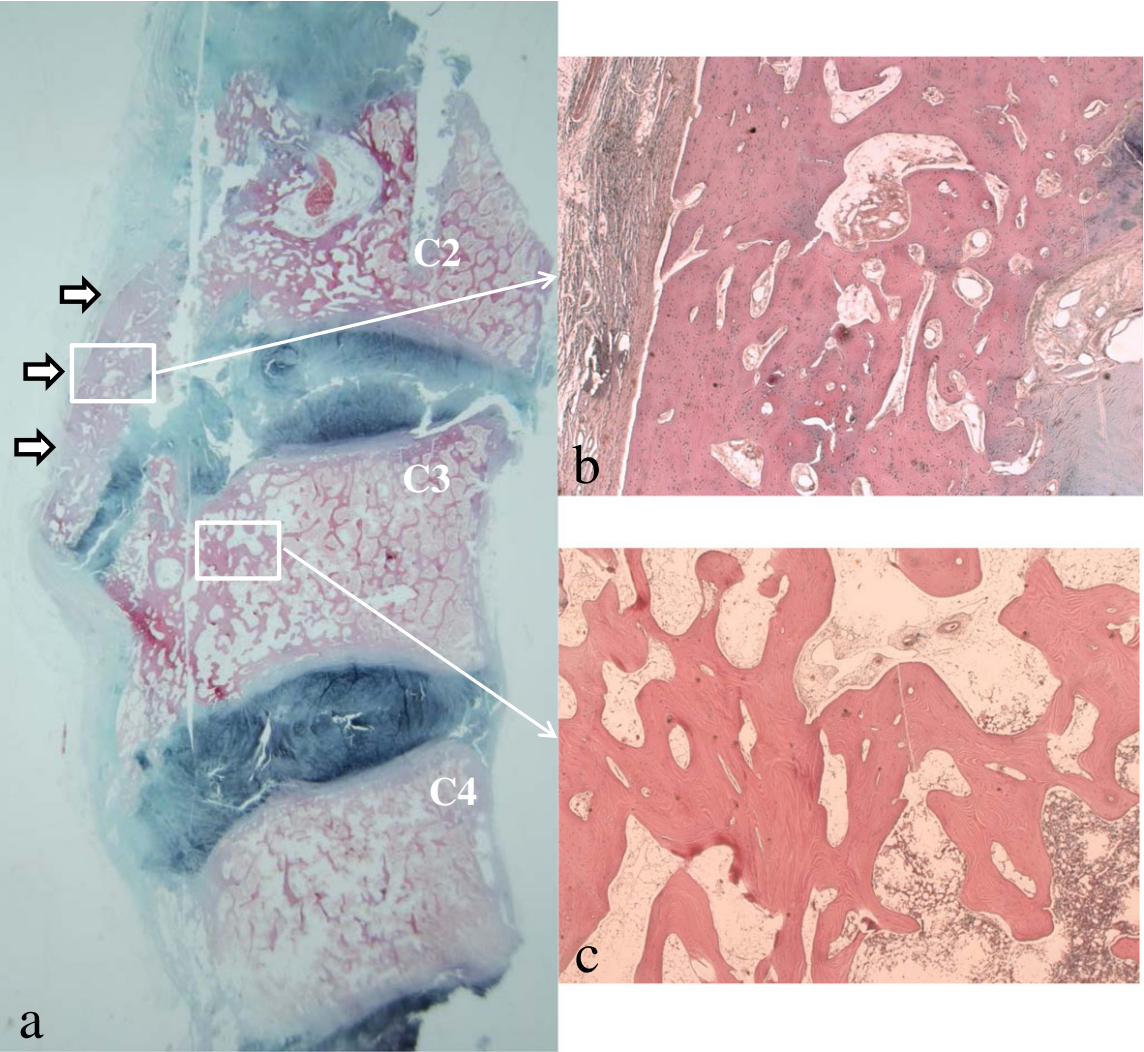
**a** Histopathological sagittal section of the removed C2 to C4 stained with Elastica-Masson revealed only a small ossification of the anterior longitudinal ligament with bone marrow (*arrows*), suggesting that etidronate disodium suppressed a regrowth of the ossification of the anterior longitudinal ligament with anterior osteophytes. **b** A high-power image (×20) of the small ossification of the anterior longitudinal ligament showed trabecular architecture. **c** The trabecular bone structure was thicker and connectivity of trabecular bones was more pronounced in the anterior and cranial regions than in the posterior region of the C3 vertebral body on a high-power image (×20)

However, he died suddenly during the night 1 year 6 months after the second surgery. The autopsy results indicated respiratory failure as the cause of death, but the details are unknown. Bone tissue was collected from the surgical site during the autopsy, and the samples were prepared for examination. Pathological findings of the small OALL included slight regeneration of a bone spur with the bone marrow, suggesting that etidronate disodium suppressed the enlargement of the matured ossification and osteophytes formation while maintaining the disc space (Fig. 4a, b) and extending across the intervertebral disc space (Fig. 4a). The trabecular bone structure was thicker and the connectivity, which is the connection of the strut-shaped trabecular bone structures, was more pronounced in the anterior and cranial regions than in the posterior region of the C3 vertebral body (Fig. 4b, c). Osteocytes were observed in the trabecular bones in the C3 vertebral body under a higher dose of etidronate treatment (Fig. 4c). These histopathological findings indicated that the etidronate therapy for OALL suppressed the enlargement of the ossification and the osteophytes formation while maintaining a normal trabecular bone structure.

## Discussion

DISH presents with ossification of the spinal column, pelvis, and attachment sites of the capsular ligament as reported by Resnick *et al*. in 1978 [7]. DISH is characterized by thickening and calcification of soft tissues, specifically the ligaments, tendons, or joint capsules, resulting in secondary formation of osteophytes. Among the paraspinal ligaments, the anterior longitudinal ligament is more severely affected than the posterior longitudinal ligament [8]. The prevalence of symptomatic OALL in patients with DISH is highest in men in their 60s [1]. The reported incidence of dysphagia due to DISH is 17 to 28 % [9]. Although conservative treatment with anti-inflammatory medication may be effective for mild dysphagia [10], OALL-induced dysphagia, which is severe or resistant to conservative treatment, should be treated by decompression of the protruding ligamentous ossification [11]. In the present case, resection of the ossification and osteophytes improved our patient’s dysphagia after the first and second surgeries. Thus, surgical resection of an OALL is obviously effective for the treatment of dysphagia caused by mechanical compression by an OALL with anterior osteophytes. However, symptomatic regrowth of the ossification and osteophytes were reported in two patients with DISH at 5.4 and 7.0 years after the initial surgical resection [12]. We prescribed etidronate to prevent the recurrence of symptomatic ossification of the ligament in the present case.

Etidronate disodium can inhibit the progression of ligament ossification in rats without any histopathological changes [5]. The histological findings before and after etidronate treatment also revealed a normal trabecular bone structure in the present case. In humans, etidronate has been shown to decrease the prevalence of heterotopic ossification in patients with total hip arthroplasty [13]. For the ossification of spinal ligament, posterior surgery such as laminectomy or laminoplasty to decompress the spinal cord has been frequently performed in patients with ossification of the posterior longitudinal ligament (OPLL) at the cervical spine. Enlargement of the remaining OPLL may occur after this posterior surgery for OPLL. Ono *et al*. [6] performed a study involving 1 year of cyclic administration of etidronate disodium to suppress postoperative enlargement of OPLL and reported that a dose of 1000 mg/day is effective for this purpose. Thus, OALL is often merged into OPLL, and the effect of etidronate on OALL should be suppression of enlargement of ossification with normal trabecular bone structure.

A previous study classified OALL into three types: type 1 (the earliest change) is characterized by a small focus of ossification on the external aspect of at least two separate vertebral bodies; type 2, in which ossified bone extends across the intervertebral disc space, forming a continuous band with the adjacent vertebral body but distinct from any annulus fibrosis calcification; and type 3, in which the ossification extends along the anterior longitudinal ligament, overlapping four or more consecutive vertebrae [14]. In the present case, our patient had a type 3 OALL at the time of the first surgery and a type 2 OALL before the second surgery (Fig. 2a) by radiographs. Furthermore, the OALL at 1.5 years after the second surgery was also type 2 (Fig. 3d) by radiograph and also by histology (Fig. 4a). We speculate that etidronate suppressed the enlargement of the ossification and the anterior osteophytes formation maintaining a normal trabecular bone structure.

Our patient received etidronate disodium at 800 mg/day for 6 months after the initial surgery to prevent recurrence of ossification. However, discontinuation of the etidronate disodium resulted in heterotopic ossification at the same site, and he required a second surgery. We performed cyclic administration of etidronate disodium at 1000 mg/day after the second OALL surgery, and no regrowth of the symptomatic ossification occurred for 1.5 years postoperatively. We consider that the dose of etidronate therapy used after the second surgery suppressed regrowth of the OALL with anterior osteophytes in the present case. There are no previous reports on the treatment of OALL using etidronate.

## Conclusions

In the present case, a regrowth of the OALL with anterior osteophytes was not observed with a 3-month cyclic administration of etidronate disodium at a dose of 1000 mg/day (3-month administration period followed by a 3-month unmedicated period) for 1 year and 5 months. To the best of our knowledge, this is the first report to describe the pathological findings of an OALL after treatment with etidronate disodium. However, further investigations are needed to clarify the effects of cyclical administration of etidronate disodium on regrowth of an OALL with anterior osteophytes.

## Abbreviations

DISH: Diffuse idiopathic skeletal hyperostosis
OALL: Ossification of the anterior longitudinal ligament
OPLL: Ossification of the posterior longitudinal ligament
